# Penile Pseudomyogenic Hemangioendothelioma: Diagnostic and Therapeutic Considerations

**DOI:** 10.7759/cureus.92852

**Published:** 2025-09-21

**Authors:** Ameer Nsair, Etan Eigner, Inbal Farkash, Valentine Shabataev, Ariel Zisman

**Affiliations:** 1 Urology, Rambam Medical Center, Haifa, ISR; 2 Pathology, Rambam Medical Center, Haifa, ISR; 3 Urology, Rambam Health Care Campus, Haifa, ISR

**Keywords:** 3d modeling, mri penis, organ-sparing surgery, pseudomyogenic hemangioendothelioma, rare vascular tumor, ­reconstructive surgery

## Abstract

Pseudomyogenic hemangioendothelioma (PHE) is a rare vascular tumor of low-grade malignancy, most commonly affecting the limbs of young males. Its occurrence in the penis is extremely uncommon. We present a case of a 19-year-old male with a one-year history of a painful penile mass. Imaging and histopathology confirmed the diagnosis of PHE localized to the right corpus cavernosum, with partial involvement of the left side. Preoperative planning included MRI with pharmacologically induced erection and 3D modeling. Reconstructive organ-sparing surgery was performed, involving excision of the midportion of the right corpora cavernosa and partial excision of the left, followed by reconstruction using a bovine pericardial graft.

At the six-month follow-up, no recurrence of the disease was observed on PET-CT, and the patient exhibited preserved urinary and erectile function, with moderate right-sided curvature. A minor revision procedure was required due to graft infection. This report highlights the diagnostic and therapeutic challenges associated with penile PHE. A multidisciplinary approach incorporating advanced imaging and 3D modeling can facilitate function-preserving surgery with satisfactory short-term outcomes.

## Introduction

Pseudomyogenic hemangioendothelioma (PHE) is a rare, low-grade vascular tumor of endothelial origin, first described by Hornick and Fletcher in 2011 [1]. It belongs to the spectrum of vascular neoplasms but is distinct in its myoid and epithelioid-like histologic pattern, which can mimic more aggressive tumors such as epithelioid sarcoma. PHE primarily affects young adult males with a mean age of 30 years, with only about 18% of patients being older than 40 years. No racial or ethnic predilection has been reported [1]. It typically presents as multiple nodules involving the superficial or deep soft tissues of the legs, and, less frequently, the arms or trunk. Unlike its microscopic mimicker, epithelioid sarcoma, PHE has an indolent course and rarely metastasizes. Long-term survival in affected patients is excellent, with disease-specific mortality being exceedingly rare [2]. In a pooled analysis of 199 cases, local recurrence was reported in about 58% of patients, often within two years of resection [3]. The five-year overall survival rate is reported to exceed 95%, emphasizing the indolent behavior of the tumor [4].

The diagnosis of PHE relies on histopathological examination with confirmatory immunohistochemistry, which remains the gold standard [1]. Imaging modalities such as MRI and CT are useful for delineating tumor extent, evaluating multifocality, and planning surgical management [5]. The treatment of choice is complete surgical excision, though local recurrence may occur, necessitating close follow-up [6]. Extra-extremity presentations are exceedingly uncommon, with only 10 cases of penile PHE described in the literature to date [7,8]. We report a case of penile PHE, emphasizing the extreme rarity of this location and the diagnostic and therapeutic challenges it presents.

## Case presentation

History and examination

**Figure 1:**
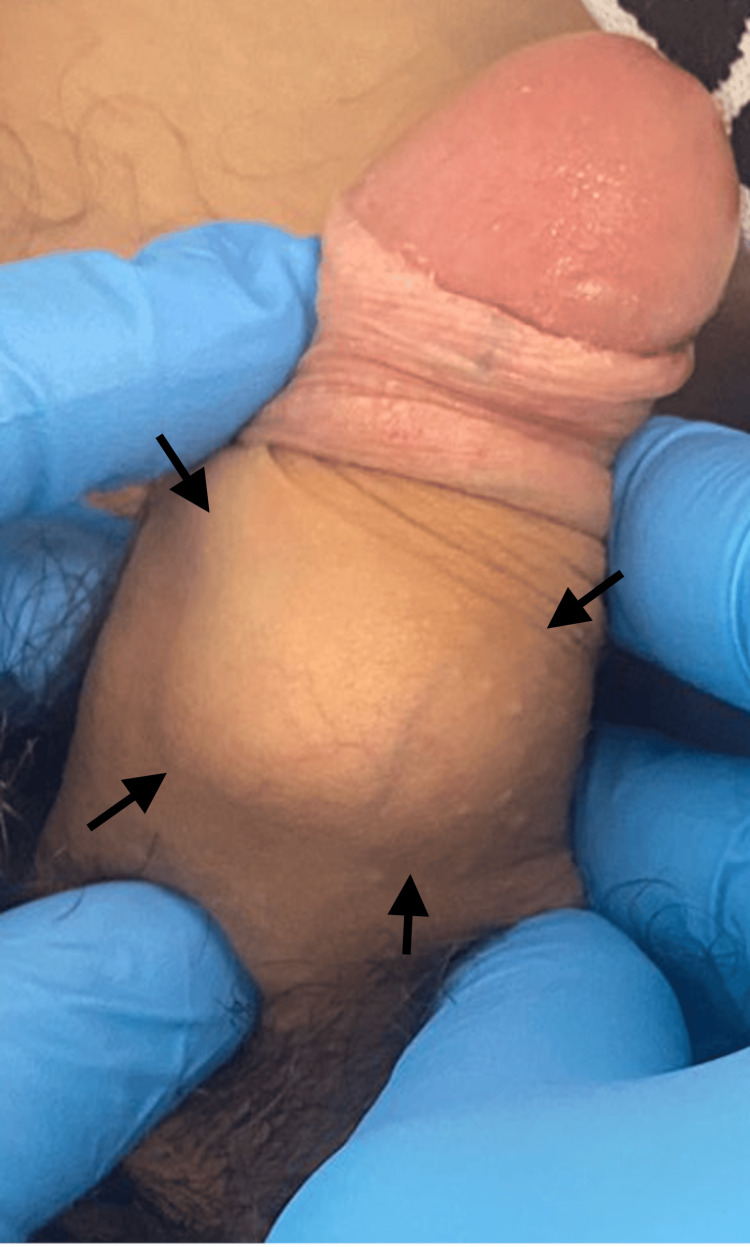
The penile solid mass at presentation

A 19-year-old male presented with a one-year history of a solid mass localized in the penis, accompanied by painful erections. His past medical history was unremarkable, and he reported no history of sexual activity or sexually transmitted diseases. There was no family history of any malignancy. On physical examination, a solid, circumferential, tender lesion was identified at the midshaft, predominantly involving the right side. The overlying skin appeared normal, with no evidence of warmth, swelling, or erythema. No palpable inguinal lymph nodes were found on examination. The presenting solid mass in the penis is exhibited in Figure 1.

Evaluation

The initial differential diagnosis for this lesion included benign or malignant neoplasms, as well as inflammatory or infectious conditions. The diagnostic workup included histopathological assessment and various imaging modalities. An ultrasound-guided fine needle transdermal core biopsy was performed. Microscopic examination revealed tumor cores composed of spindle, epithelioid, and vacuolated cells with minimal atypia. No necrosis was observed. These findings were consistent with PHE.

MRI exhibited a lesion measuring 2.1 × 2.0 × 3.4 cm in the midshaft of the penis. The lesion primarily affected the right corpus cavernosum and partially extended into the left corpus cavernosum. It disrupted the tunica of both corpora and infiltrated the subcutaneous tissue while sparing the corpus spongiosum and urethra. Small lymph nodes were identified in the groin and external iliac nodes bilaterally. However, no evidence of metastatic disease was noted.

**Figure 2:**
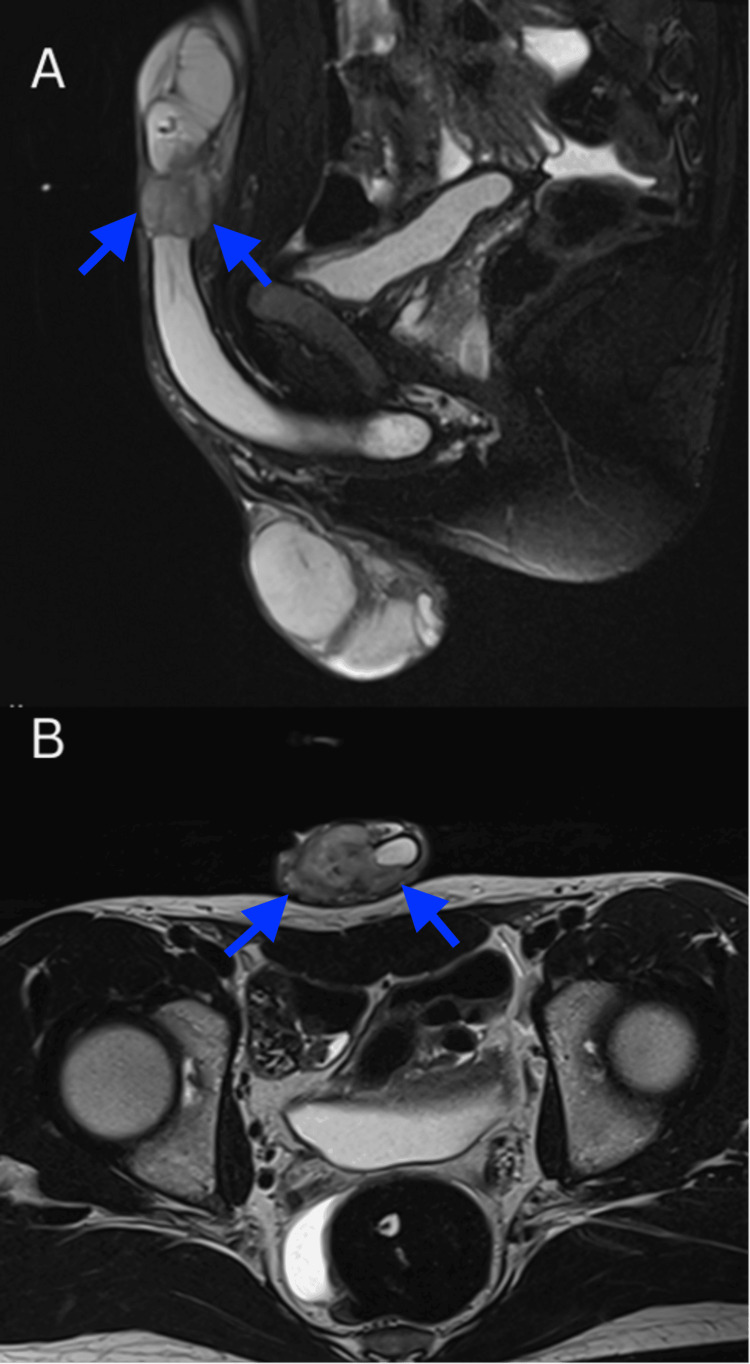
MRI with pharmacologically induced erection A. Sagittal MRI showing a penile mass involving the right corpus cavernosum. B. Axial slide exhibiting the partial extension to the left corpus cavernosum with sparing of the urethra MRI: magnetic resonance imaging

To better understand the anatomical borders of the lesion and assist in surgical planning, a second MRI was performed with a pharmacologically induced erection. This was achieved by intracavernosal injection of alprostadil. The second MRI revealed that the lesion involved the entire circumference of the tunica of the right corpus cavernosum, with partial penetration into the left corpus cavernosum, but sparing the urethra. Both dorsal penile arteries and the right cavernous artery were also involved. Sagittal and axial MRI slides of the lesion are presented in Figure 2.

**Figure 3:**
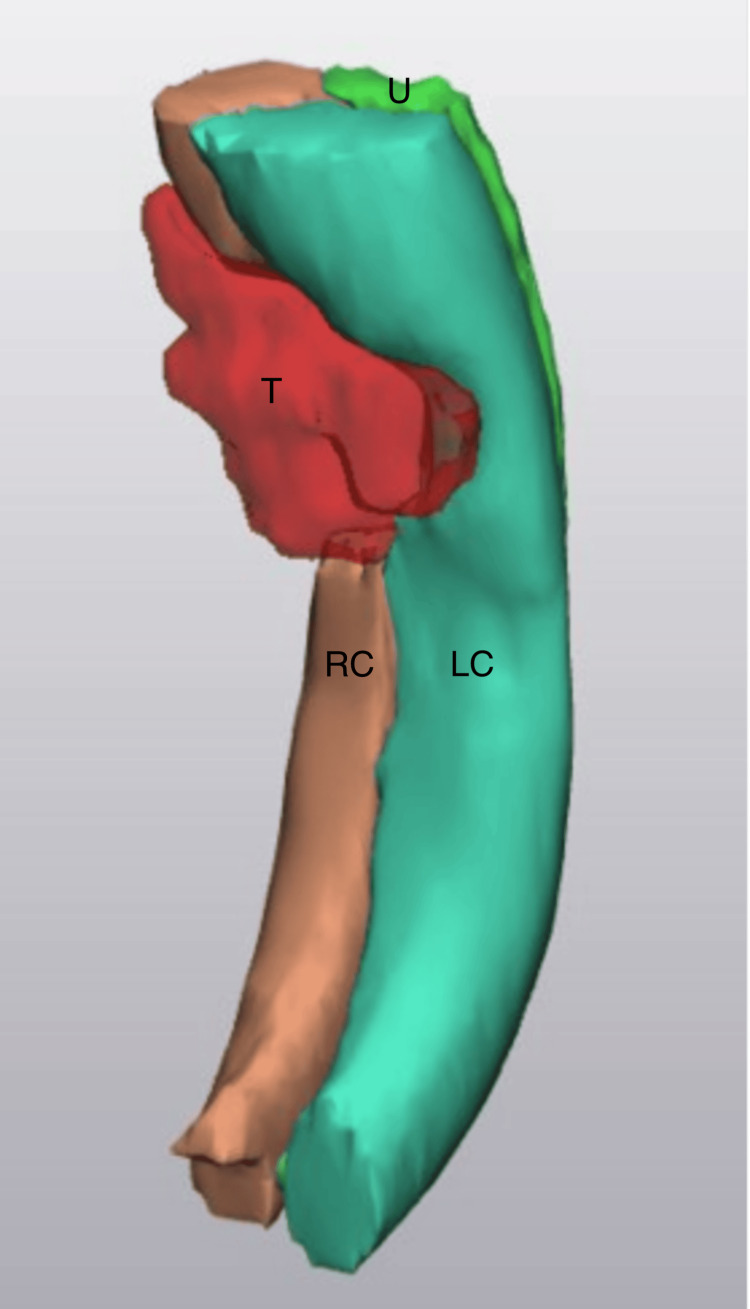
3D-printed model of the lesion and surrounding anatomy, used for surgical planning T: tumor; RC: right corpora cavernosa; LC: left corpora cavernosa; U: urethra

PET-CT demonstrated increased fluorodeoxyglucose (FDG) uptake in the penile lesion, with no evidence of distant metastasis. For 3D visualization, CT images were segmented using Mimics Innovation Suite, version 21.0 (Materialise NV, Leuven, Belgium). The resulting anatomical regions were then exported to 3-matic software (Materialise Inc.) for post-processing. The final model was printed using a fused deposition modeling (FDM) printer with poly(lactic acid) (PLA) material. A three-dimensional reconstruction of the penile lesion is shown in Figure 3.

Surgical procedure

After carefully considering all factors, including the patient's young age and the tumor's low malignant potential with a low risk of metastasis, we decided to proceed with organ-sparing reconstructive surgery. Our primary goal was complete tumour resection while aiming to preserve erectile function, maintain the ability to void while standing, and achieve the best possible aesthetic outcome.

**Figure 4:**
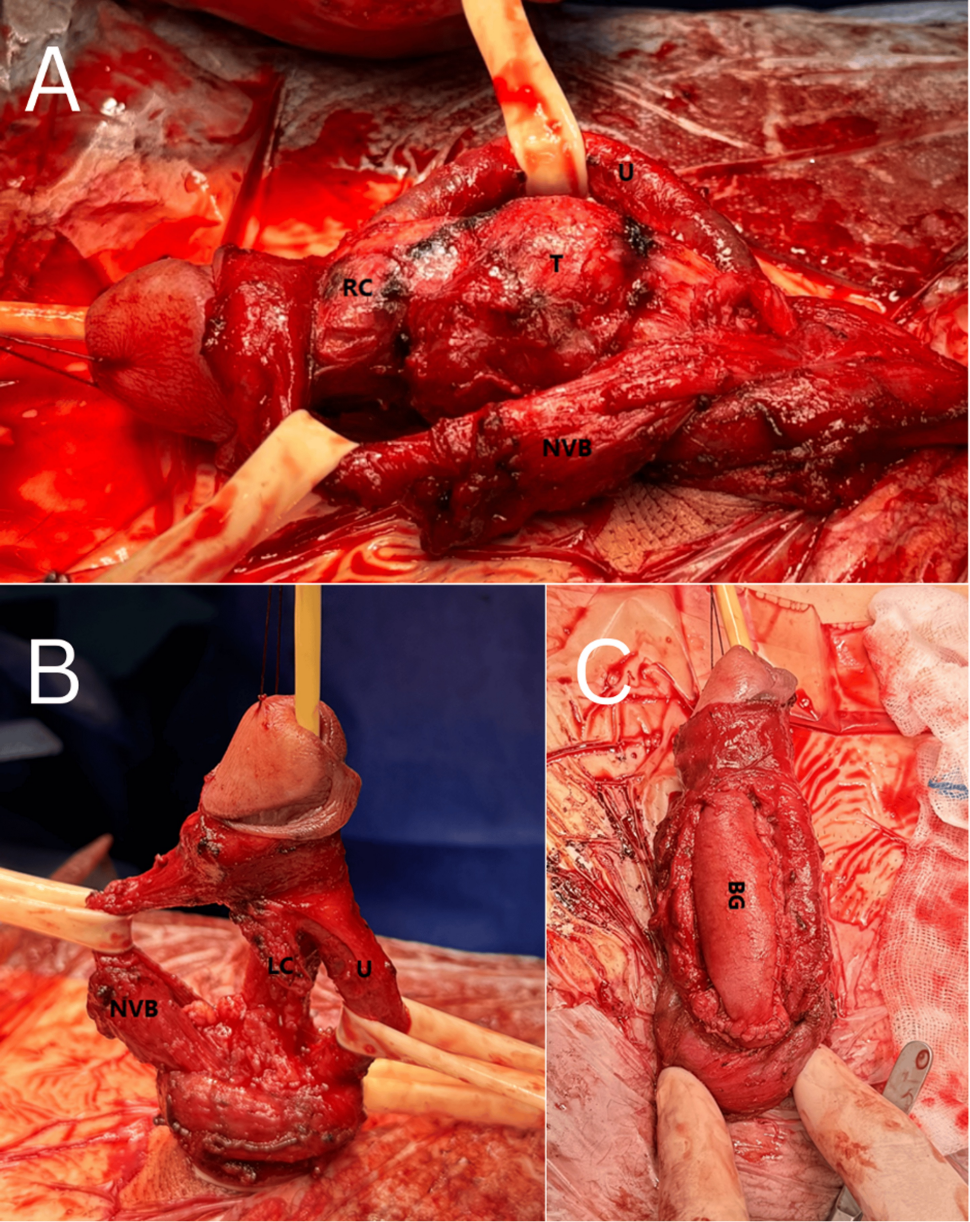
Surgical procedure A. Retracted neurovascular bundle and urethra during dissection. B. After resection of the right corpora and partial excision of the left, with preservation of the urethra and neurovascular bundle. C. Final reconstruction using a bovine pericardial patch U: urethra; NVB: neurovascular bundle; RC: right corpora cavernosa; LC: left corpora cavernosa; T: tumor; BG: bovine pericardial graft

After degloving the penis, we carefully identified the neurovascular bundle. We were able to meticulously dissect and retract most of it away from the lesion (Figure 4A). The corpus spongiosum and the urethra were identified and retracted as well. The lesion appeared to compress the urethra but showed no signs of invasion. The mass was excised from both corpora, and frozen sections from the margins of the defect were sent for pathological evaluation. Additional dissection was performed at sites with positive margins until clear margins were confirmed. As a result, the midportion of the right corpus cavernosum was removed en bloc with the mass, along with approximately 70% of the left corpus cavernosum. Despite the extensive resection, both the urethra and the neurovascular bundle were successfully preserved (Figure 4B). To reconstruct the defect in the right corpora, a bovine pericardial patch (TisGenX™, TisGenX Medical, Irvine, CA) was sutured with permanent 2-0 Ethibond sutures (Figure 4C).

Pathological evaluation

**Figure 5:**
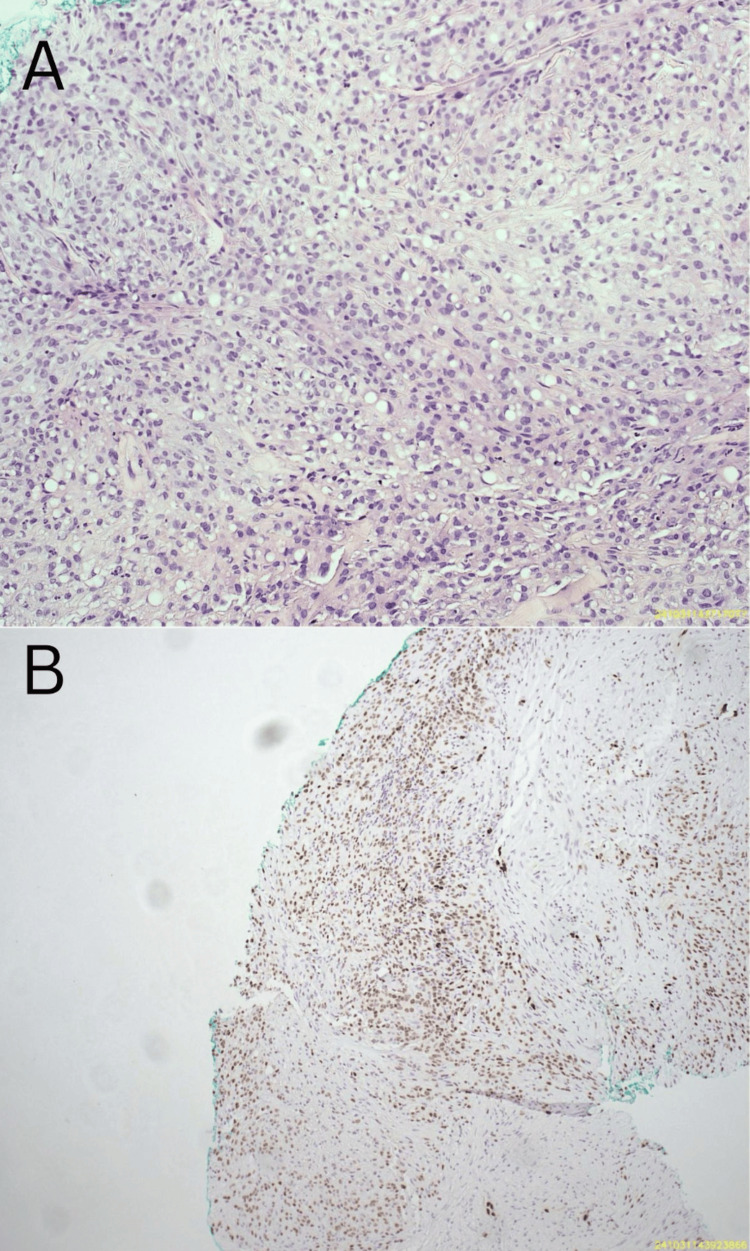
Microscopic evaluation A. Spindle cells with an eosinophilic cytoplasm and cytoplasmic vacuolization. B. Vascular nature of the tumor verified by ERG positivity

Gross examination revealed a tumor measuring 2 cm in diameter, characterized by gray nodules with ill-defined margins and an infiltrative growth pattern. Microscopic evaluation demonstrated a multinodular proliferation of spindle cells containing eosinophilic cytoplasm with notable cytoplasmic vacuolization (Figure 5A). The neoplastic cells exhibited moderate nuclear atypia, with a mitotic rate of 1-2 mitoses per high-power field. Immunohistochemical staining demonstrated positivity for keratin, ETS-related gene, Friend leukemia integration 1 transcription factor, and FosB proto-oncogene, while calmodulin-binding transcription activator 1 was negative (Figure 5B). Integrase interactor 1 expression was retained in the tumor cells.

Follow-up

The patient required only analgesics during the perioperative phase and was discharged on oral amoxicillin-clavulanate (Augmentin) for one week. During the follow-up, the patient had normal urination and preserved erections but with moderate right-sided curvature. Two months post-op, the bovine pericardial graft showed signs of infection and eroded to the skin. The infected graft was completely removed. At the six-month follow-up, the area had epithelialized completely. The patient maintained both erectile function and the ability to void while standing. PET-CT revealed no evidence of residual disease or ongoing infection.

## Discussion

This case represents a highly unusual presentation of PHE in the penis of a young male, posing several diagnostic and therapeutic challenges. PHE predominantly affects the extremities, and only 10 penile cases have been previously described in the literature to date [7,8]. Ide et al. reported a solitary lesion in a 43-year-old man [7], while Youssef et al. documented five additional cases together with a review of five earlier reports, noting variation in tumor extent and treatment approaches [8]. Our patient similarly presented with a solitary lesion amenable to organ-sparing surgery, but with unique features that distinguish this case: full involvement of one corpus cavernosum with partial extension into the contralateral side, evaluation with novel diagnostic strategies (MRI with pharmacologically induced erection and 3D modeling), and reconstruction using a graft following partial corporeal resection. These aspects underscore both the diagnostic and therapeutic challenges posed by this rare tumor location and highlight the innovation required in its management. Given the rarity of this presentation, combined with its histological overlap with other soft tissue tumors, a thorough diagnostic workup is mandatory. In this context, immunohistochemistry was essential to differentiate PHE from more aggressive tumors such as epithelioid sarcoma.

MRI with pharmacologically induced erection, previously shown to enhance local staging in penile cancer by delineating corporal involvement [9], proved particularly valuable in this case of a low-malignant-potential tumor such as PHE. In the original study, MRI was effective in identifying tumor infiltration into the corpora cavernosa or corpus spongiosum, which are key factors in distinguishing between T1-T2 and T3-T4 stages. In some patients, MRI findings led to a modification of the surgical approach, such as opting for a more conservative or more extensive resection than initially planned based on physical examination alone. Thus, MRI with artificial erection was especially valuable in borderline or ambiguous cases, helping to avoid both overtreatment and undertreatment. In our case, it provided precise mapping of tumor borders, delineated the extent of corpus cavernosum involvement bilaterally as well as the sparing of the urethra, and clarified vascular proximity. Importantly, these findings directly influenced the surgical plan, allowing us to proceed with organ-sparing excision, anticipate the required reconstruction, and select the appropriate graft.

3D printing has shown increasing value in urology for enhancing anatomical visualization and surgical planning. 3D models have been shown to aid in planning partial nephrectomies and other anatomically demanding procedures by reducing operative time and improving intraoperative decision-making [10]. For rare tumor cases, the ability to render medical images into a physical model can significantly bolster the spatial comprehension of the lesion and assist in developing more accurate surgical strategies, with a focus on preserving the affected organs. To our knowledge, the integration of 3D modeling and printing in preoperative planning for penile PHE is novel and was instrumental in planning a functional and aesthetic reconstruction.

A multidisciplinary discussion was key in developing a treatment strategy. Given the tumor's low malignant potential and absence of validated neoadjuvant approaches, we opted for organ-sparing surgical excision with intraoperative frozen sections. Reconstruction was performed using a bovine pericardium graft, adapted from the plaque excision and grafting technique for Peyronie’s disease [11]. Although the graft eventually became infected, the complication was managed with a minor revision procedure, yielding acceptable functional and aesthetic outcomes. Contributing factors likely included the graft’s exposure to a naturally colonized environment, prolonged operative time, and local tissue handling. Prevention strategies in analogous reconstructive contexts - such as penile prosthesis surgery - include meticulous intraoperative technique (e.g., the “no-touch” method), antibiotic-coated grafts or devices, perioperative antibiotic prophylaxis, and thorough skin preparation with chlorhexidine-based solutions [12-14]. Raising awareness of this potential complication is crucial for future cases, as prompt recognition and timely surgical revision can preserve both function and cosmesis.

This report involves a single-patient case with short follow-up, and hence its findings may not be generalizable for long-term recurrence; however, it underscores the importance of personalized, multidisciplinary care in rare penile tumors. It also highlights the emerging role of advanced imaging and surgical planning tools in their management.

## Conclusions

Penile PHE is a rare diagnosis requiring a histopathologic confirmation. This report highlights the role of advanced imaging and organ-sparing reconstruction. Longer follow-up and additional case reports, ideally through multicentre collaborations, are needed to define standardized management and analyze long-term outcomes.

## References

[1] Hornick JL, Fletcher CDM (2011). Pseudomyogenic hemangioendothelioma: a distinctive, often multicentric tumor with indolent behavior. Am J Surg Pathol.

[2] Horan NA, DiMaio DJ (2017). Pseudomyogenic hemangioendothelioma. Cutis.

[3] Yang N, Huang Y, Yang P, Yan W, Zhang S, Li N, Feng Z (2023). Clinicopathological study of pseudomyogenic hemangioendothelioma. Diagn Pathol.

[4] Mosquera C, Argani P, Pietanza MC (2020). Clinical outcomes of patients with pseudomyogenic hemangioendothelioma: A multi-institutional study. Mod Pathol.

[5] Cioffi A, Italiano A, Penel N (2013). Pseudomyogenic hemangioendothelioma: a clinicopathologic study of 5 cases, including one with multifocal intramuscular involvement. Skeletal Radiol.

[6] McGinity M, Bartanusz V, Dengler B, Birnbaum L, Henry J (2013). Pseudomyogenic hemangioendothelioma (epithelioid sarcoma-like hemangioendothelioma, fibroma-like variant of epithelioid sarcoma) of the thoracic spine. Eur Spine J.

[7] Ide YH, Tsukamoto Y, Ito T (2015). Penile pseudomyogenic hemangioendothelioma/epithelioid sarcoma-like hemangioendothelioma with a novel pattern of SERPINE1-FOSB fusion detected by RT-PCR--report of a case. Pathol Res Pract.

[8] Youssef R, Davis JL, Anderson WJ, Acosta AM (2024). Pseudomyogenic hemangioendothelioma presenting as a penile lesion. Virchows Arch.

[9] Scardino E, Villa G, Bonomo G (2004). Magnetic resonance imaging combined with artificial erection for local staging of penile cancer. Urology.

[10] Erman A, Masucci L, Krahn MD, Elterman DS (2018). Pharmacotherapy vs surgery as initial therapy for patients with moderate-to-severe benign prostate hyperplasia: a cost-effectiveness analysis. BJU Int.

[11] Otero JR, Gómez BG, Polo JM (2017). Use of a lyophilized bovine pericardium graft to repair tunical defect in patients with Peyronie's disease: experience in a clinical setting. Asian J Androl.

[12] Matthew-Onabanjo AN, Matthew AN, Famati E, Nguyen V, Rogers MJ (2024). Perioperative infection prevention during inflatable penile prosthesis surgery: a narrative review. Transl Androl Urol.

[13] Best JC, Clavijo RI (2020). Best practices for infection prevention in penile prosthesis surgery. Curr Opin Urol.

[14] Carrasquillo RJ, Gross MS (2018). Infection prevention strategies prior to penile implant surgery. Eur Urol Focus.

